# Low-temperature CO oxidation over Cu/Pt co-doped ZrO_2_ nanoparticles synthesized by solution combustion

**DOI:** 10.3762/bjnano.8.156

**Published:** 2017-07-31

**Authors:** Amit Singhania, Shipra Mital Gupta

**Affiliations:** 1 Department of Chemical Engineering, Indian Institute of Technology Delhi, Hauz Khas, New Delhi 110016, India; 2 University School of Basic and Applied Sciences, Guru Gobind Singh Indraprastha University, Sector 16-C, Dwarka, New Delhi 110078, India

**Keywords:** CO oxidation, copper, nanoparticles, platinum, solution combustion, zirconia

## Abstract

Zirconia (ZrO_2_) nanoparticles co-doped with Cu and Pt were applied as catalysts for carbon monoxide (CO) oxidation. These materials were prepared through solution combustion in order to obtain highly active and stable catalytic nanomaterials. This method allows Pt^2+^ and Cu^2+^ ions to dissolve into the ZrO_2_ lattice and thus creates oxygen vacancies due to lattice distortion and charge imbalance. High-resolution transmission electron microscopy (HRTEM) results showed Cu/Pt co-doped ZrO_2_ nanoparticles with a size of ca. 10 nm. X-ray diffraction (XRD) and Raman spectra confirmed cubic structure and larger oxygen vacancies. The nanoparticles showed excellent activity for CO oxidation. The temperature *T*_50_ (the temperature at which 50% of CO are converted) was lowered by 175 °C in comparison to bare ZrO_2_. Further, they exhibited very high stability for CO reaction (time-on-stream ≈ 70 h). This is due to combined effect of smaller particle size, large oxygen vacancies, high specific surface area and better thermal stability of the Cu/Pt co-doped ZrO_2_ nanoparticles. The apparent activation energy for CO oxidation is found to be 45.6 kJ·mol^−1^. The CO conversion decreases with increase in gas hourly space velocity (GHSV) and initial CO concentration.

## Introduction

The catalytic oxidation of carbon monoxide (CO) is of potential interest in applications such as CO sensors, carbon dioxide (CO_2_) lasers, cigarettes, proton-exchange membrane fuel cells, air purifiers, methanol production and water-gas shift reaction [1–4]. The catalytic oxidation of CO was revolutionized by Haruta et al. [5]. They worked on supported nanogold catalysts and found them to be highly active for CO oxidation. Till date, different types of catalysts including monometallic (e.g., Pt, Pd, Rh, Au, Ni, Co and Sn), bimetallic (e.g., Pd–Au, Pd–Rh, Pt–Co, Cu–Rh, Au–Cu and Au–Ag) along with various types of supports (e.g., CeO_2_, SiO_2_, Al_2_O_3_, Co_3_O_4_, Fe_2_O_3_, activated carbon (AC), carbon nanotubes (CNTs) and ZrO_2_) have been reported for CO oxidation reaction in literature [6–12]. Recently, ZrO_2_ has been used as a catalyst and support in different catalytic reactions such as solid-oxide fuel cells, ethanol reforming, hydrogen generation and hydrogenation [13–17]. It is reported to be more inert in acidic reaction environments [18] and a better catalyst/support than other materials such as SiO_2_, TiO_2_, and Al_2_O_3_ [19]. Recently, we reported the usage of ZrO_2_ and Pt-doped ZrO_2_ nanoparticles for CO oxidation [20]. In this paper, the synthesized ZrO_2_ showed 100% CO conversion at a temperature which is the lowest reported so far for bare ZrO_2_. Also, the addition of Pt resulted in an increase in oxygen vacancies (oxygen source for CO oxidation reaction) and hence an increase in the efficiency of CO conversion. Cu is known as a highly active metal catalyst for CO oxidation [21–22]. Cu supported on a ZrO_2_-based composite showed very high catalytic activity [23]. The addition of Pt, Ni, Rh and Cu into the support result in an increase in oxygen vacancies, oxygen storage capacity, smaller particle size, high specific surface area, and better stability of the material [24–28]. Recently, Zheng et al. [27] reported that the addition of Cu to the support resulted in a high catalytic activity for CO oxidation. They reported a value of the temperature *T*_50_ (the temperature at which 50% CO conversion is achieved) of around 75 °C for Cu-containing catalysts. Similarly, Yang et al. [29] also reported a high activity of Cu-containing ZrO_2_-based nanocomposites (*T*_50_ = 70 °C) for CO oxidation. So, the combination of Cu, Pt and ZrO_2_ appears to be very interesting and promising.

In literature, different methods have been used for the synthesis of doped ZrO_2_ nanoparticles. These include sol–gel, ball milling, precipitation, combustion, and reverse microemulsion [30–33]. Vahidshad et al. [34] synthesized sol–gel-derived Cu–ZrO_2_ nanoparticles. Similarly, Saha et al. [35] prepared CuO-doped ZrO_2_ nanoparticles via ball milling. Among the described methods, solution combustion is used frequently due to its ability to provide high purity, highly active and stable products in very short time.

In this work, we synthesized highly active and stable Cu/Pt co-doped ZrO_2_ nanoparticles by using solution combustion and explored it for CO oxidation. To the best of our knowledge, this is the first time Cu/Pt co-doped ZrO_2_ has been used for CO oxidation.

## Results and Discussion

### Materials characterization

The BET method using nitrogen as adsorbate was employed to calculate the specific surface area and pore volume of Pt(1%)–Cu(1%)–ZrO_2_ (the numerals indicate mol %) nanoparticles. Table 1 shows a specific surface area of 65.1 m^2^·g^−1^ for Pt(1%)–Cu(1%)–ZrO_2_. The pore volume of Pt(1%)–Cu(1%)–ZrO_2_ is found to be 0.088 × 10^−6^ m^3^·g^−1^. The lattice distortion is expected here due to incorporation of Cu and Pt into ZrO_2_. This is also confirmed by a decrease in crystallite size and lattice constants values in comparison to those reported for ZrO_2_ and Pt-doped ZrO_2_ [20].

**Table 1:** Properties of synthesized Pt(1%)–Cu(1%)–ZrO_2_.

Pt^a^ (mol %)	Cu^a^ (mol %)	specific surface area^b^ (m^2^ g^-1^_)_	pore volume^c^ ×10^−6^ (m^3^·g^−1^)	crystallite size^d^ (nm)	lattice constant^e^ (Å)

0.96	0.95	65.1	0.088	10.1	5.0994

^a^material composition was determined by ICP-AES; ^b^BET surface area; ^c^total pore volume; ^d^calculated using Scherrer equation for the (111) plane; ^e^calculated using Bragg’s Law for the (111) plane.

The powder XRD diffraction patterns of pure ZrO_2_, Pt(1%)–ZrO_2_, and Pt(1%)–Cu(1%)–ZrO_2_ are shown in Figure 1. All diffraction patterns showed cubic ZrO_2_ with sharp peaks at 30.2°, 35.1°, 50.4°, 59.9° and 62.9° corresponding to the (111), (200), (220), (311) and (222) planes, respectively (JCPDS card no. 27-0997). Peaks were found neither at 2θ = 39.8° for Pt (or 2θ = 33.9° and 27.9° for PtO and PtO_2_) nor at 2θ = 43.6° for Cu (or 2θ = 38.7° and 36.5° for CuO and Cu_2_O). This indicates that Cu^2+^ and Pt^2+^ ions have entered the ZrO_2_ lattice. The Scherrer equation was used to determine crystallite size, *D*, of Pt(1%)–Cu(1%)–ZrO_2_ material:

[1]
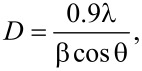


where β is the full-width at half-maximum (FWHM) in radians, λ is the used X-ray wavelength and θ is the Bragg angle.

**Figure 1:**
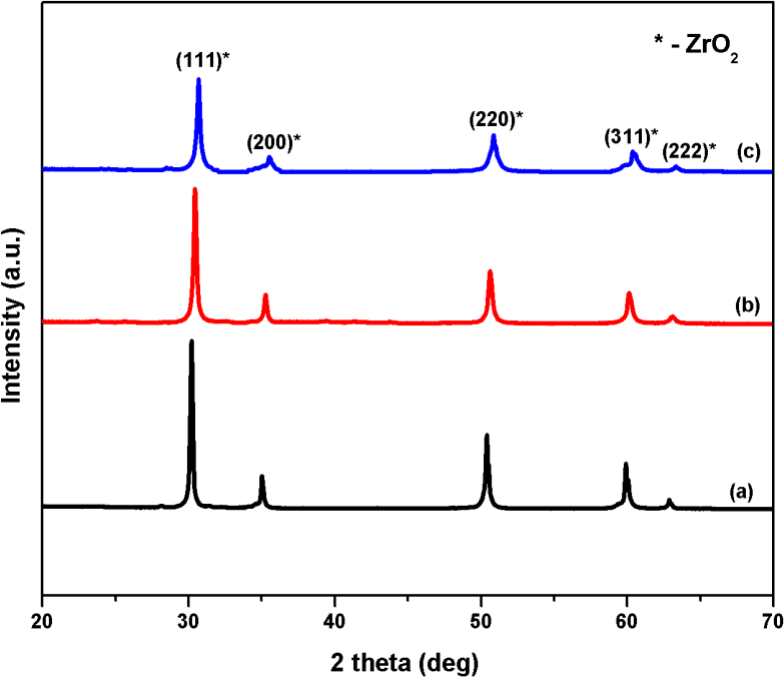
Powder XRD pattern of (a) ZrO_2_, (b) Pt(1%)–ZrO_2_, and (c) Pt(1%)–Cu(1%)–ZrO_2_.

The crystallite size of the Pt(1%)–Cu(1%)–ZrO_2_ material was calculated to be 10.1 nm by using only the reflections of the (111) planes. Figure 2 shows an expanded region of the XRD patterns. The FWHM values are increased in Pt(1%)–Cu(1%)–ZrO_2_ in comparison to pure ZrO_2_ and Pt(1%)–ZrO_2_ which indicates smaller crystallite sizes. The peaks are shifted towards higher angles indicating a smaller lattice parameter.

**Figure 2:**
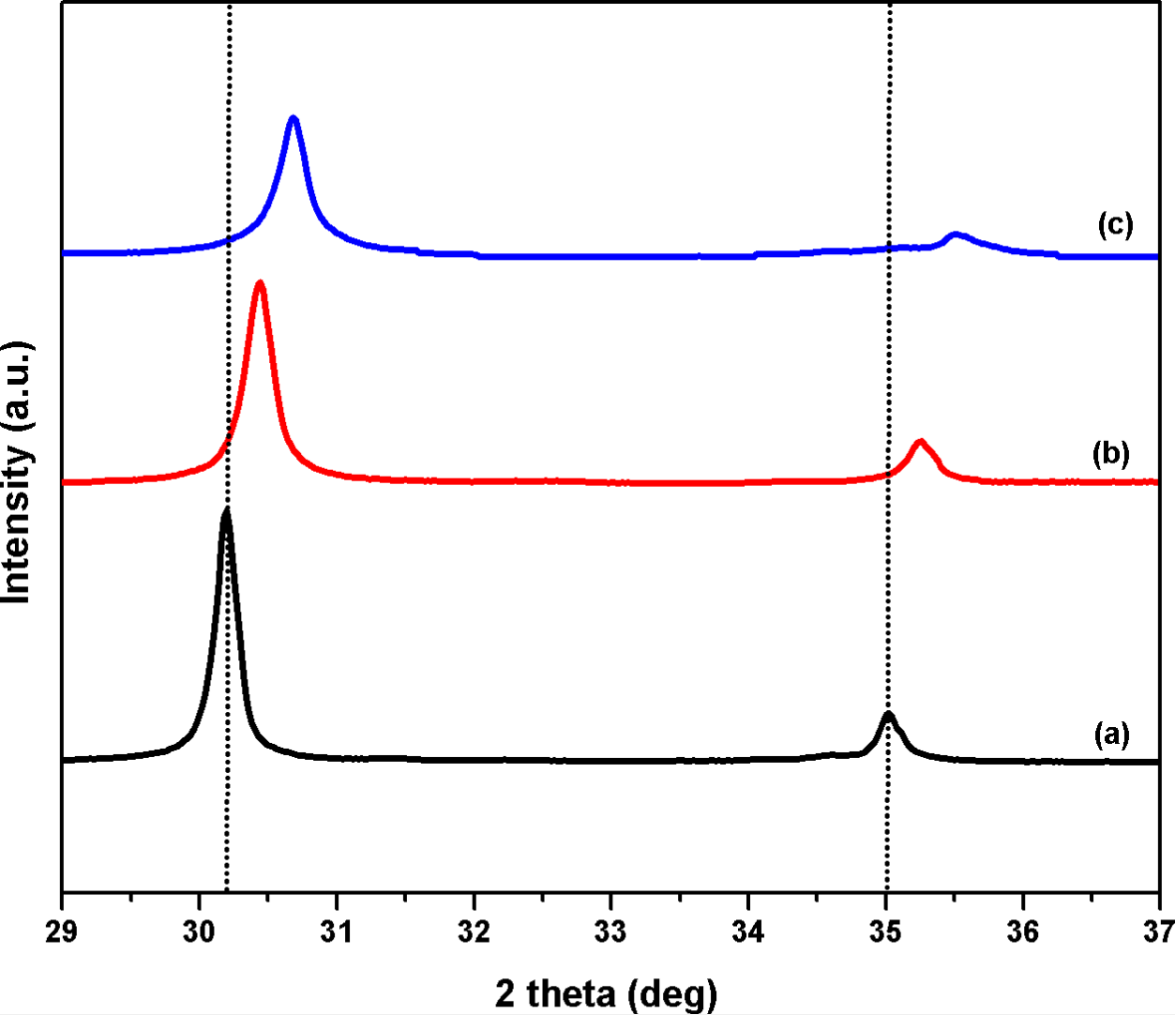
Expanded region of XRD between 29° and 37° of (a) ZrO_2_, (b) Pt(1%)–ZrO_2_ and (c) Pt(1%)–Cu(1%)–ZrO_2_.

The ionic radius of Zr^4+^ (0.86 Å) is larger than both that of Cu^2+^ (0.73 Å) and that of Pt^2+^ (0.80 Å), which resulted in a decrease of the lattice parameter from 5.1250 Å [20] to 5.0994 Å on the addition of Cu and Pt. This showed that during solution combustion Cu^2+^ and Pt^2+^ ions dissolve into the ZrO_2_ lattice. Due to the smaller ionic radii of Cu^2+^ and Pt^2+^, the structure of ZrO_2_ lattice shrinks during replacement of Zr^4+^ by Cu^2+^ and Pt^2+^ ions. This results in the generation of oxygen vacancies (source of oxygen in CO oxidation) and confirms the co-doping of Cu and Pt into ZrO_2_. As a result of this, a synergic effect is introduced between Cu, Pt and Zr components.

Figure 3 showed HRTEM analysis of Pt(1%)–Cu(1%)–ZrO_2_ nanoparticles. After addition of Cu and Pt into ZrO_2_, smaller particle sizes in comparison to ZrO_2_ and Pt(1%)–ZrO_2_ [20] were measured. The Pt(1%)–Cu(1%)–ZrO_2_ particles showed an average size of 10.7 nm. These results are similar to those obtained in XRD (Table 2).

**Figure 3:**
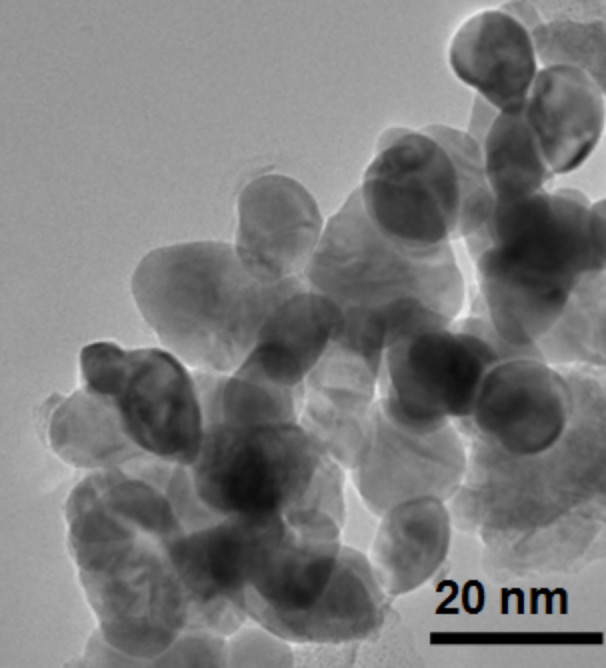
HRTEM micrograph of Pt(1%)–Cu(1%)–ZrO_2_.

**Table 2:** Calculated particle size of Pt(1%)–Cu(1%)–ZrO_2_ using XRD and HRTEM analysis.

XRD (crystallite size) (nm)	HRTEM (average particle size) (nm)

10.1	10.9

Figure 4 shows a TGA measurement of the synthesized Pt(1%)–Cu(1%)–ZrO_2_ nanoparticles in an inert atmosphere. No mass loss is seen in the tested temperature range (100–750 °C).

**Figure 4:**
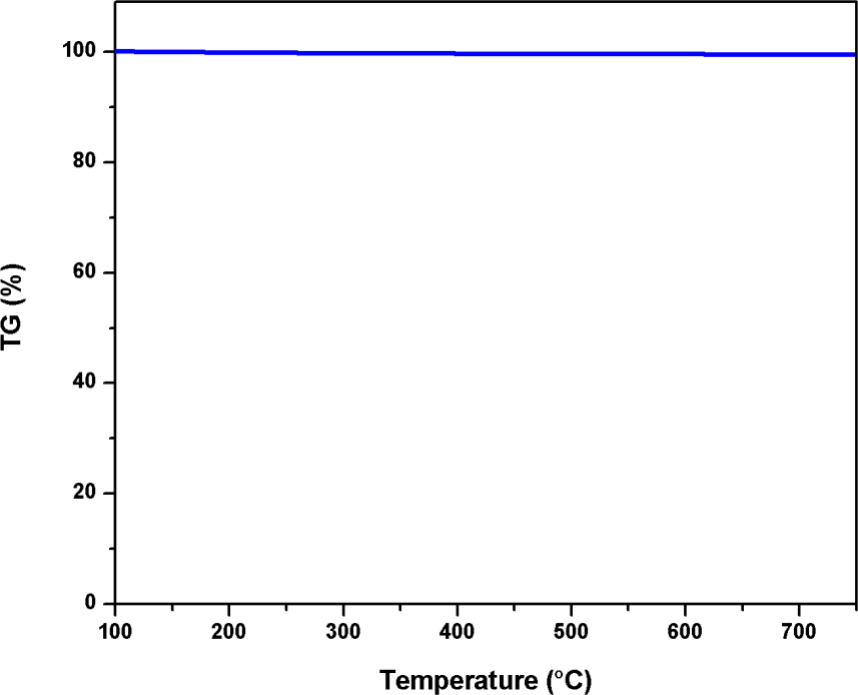
TGA measurement of Pt(1%)–Cu(1%)–ZrO_2_.

The Raman spectra of prepared ZrO_2_, Pt(1%)–ZrO_2_, and Pt(1%)–Cu(1%)–ZrO_2_ nanoparticles are shown in Figure 5. ZrO_2_ shows a strong peak at 611 cm^−1^ and two small peaks at 151 and 242 cm^−1^ corresponding to its cubic structure [36]. Incorporation of Cu and Pt into ZrO_2_ lattice broadens the strong peak and shifts it to 622 cm^−1^. This shift indicates an increase in oxygen vacancies in Pt(1%)–Cu(1%)–ZrO_2_ [37–38]. Also, the large FWHM indicates smaller sizes of the nanoparticles compared to ZrO_2_ and Pt(1%)–ZrO_2_ [39].

**Figure 5:**
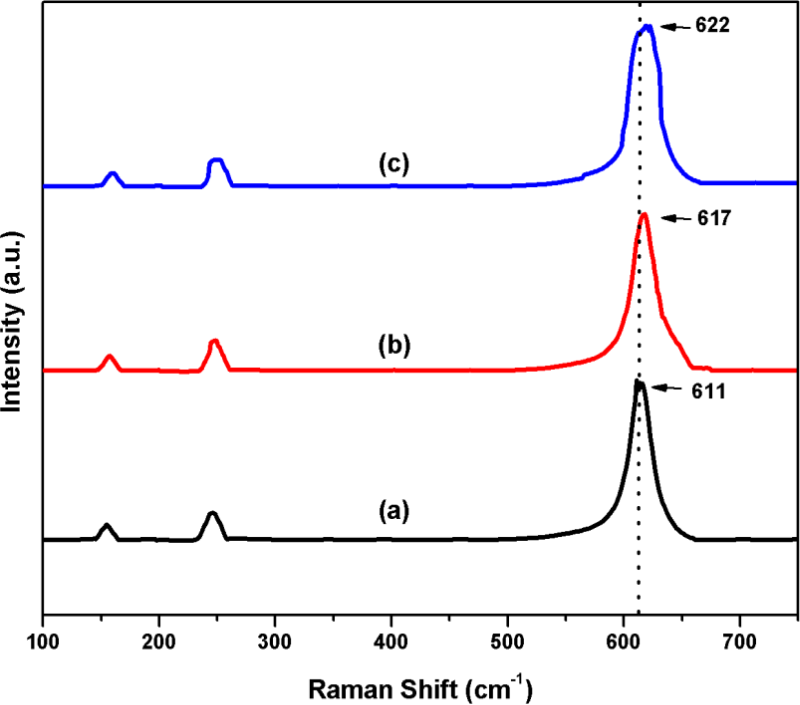
Raman spectra of (a) ZrO_2_, (b) Pt(1%)–ZrO_2_, and (c) Pt(1%)–Cu(1%)–ZrO_2_.

### Catalytic activity

Oxidation of CO was carried out with pure ZrO_2_, Pt(1%)–ZrO_2_ and Pt(1%)–Cu(1%)–ZrO_2_ nanoparticles as catalysts at different temperatures (Figure 6). The CO conversion increases with increasing temperature. The obtained values for *T*_50_ were 195 °C for ZrO_2_ and 45 °C for Pt(1%)–ZrO_2_ nanoparticles. This CO conversion was further improved by the incorporation of both Cu and Pt into the ZrO_2_ lattice. Pt(1%)–Cu(1%)–ZrO_2_ showed a *T*_50_ value of 20°C, which is a large improvement. This results can be related to the characterization results, which showed a high specific surface area, smaller particle size and larger oxygen vacancies of Pt(1%)–Cu(1%)–ZrO_2_ compared to ZrO_2_ and Pt(1%)–ZrO_2_ [20] and consequently resulted in a higher catalytic activity.

**Figure 6:**
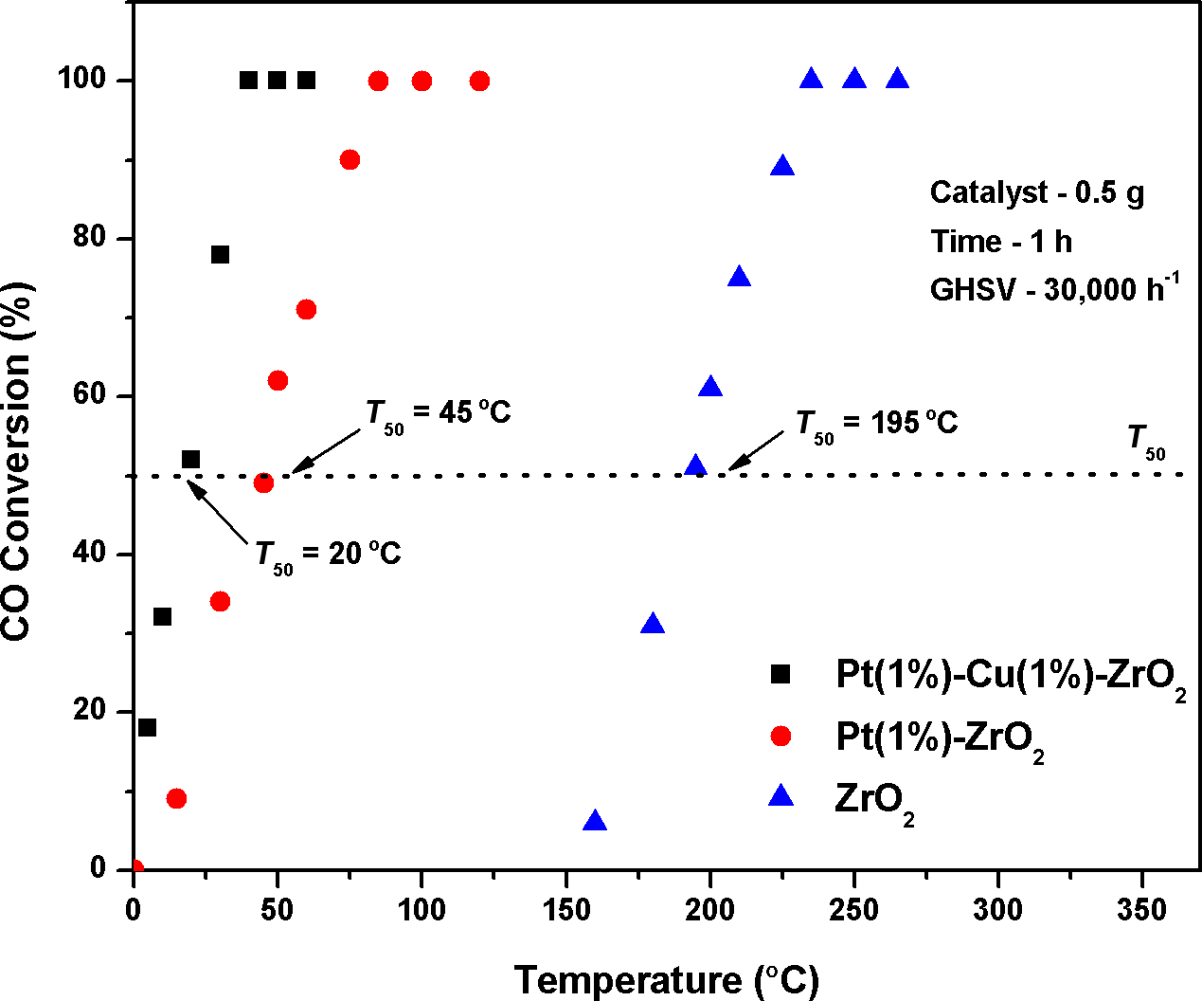
CO conversion for pure ZrO_2_, Pt(1%)–ZrO_2_, and Pt(1%)–Cu(1%)–ZrO_2_ nanoparticles (catalyst: 0.5 g, CO: 500 ppm, O_2_: 20% with Ar balance, GHSV = 30,000 h^−1^).

### Stability of Pt(1%)–Cu(1%)–ZrO_2_

Figure 7 shows the stability test of Pt(1%)–Cu(1%)–ZrO_2_ nanoparticles during the CO oxidation reaction (time-on-stream: ca. 70 h). In this study, 0.5 g of catalytic material was used and temperature of 35 °C was maintained in the vertical fixed-bed reactor with a GHSV of 30,000 h^−1^. The CO reaction results showed a constant CO conversion during the entire time-on-stream of 70 h. This confirmed the excellent stability of Pt(1%)–Cu(1%)–ZrO_2_ nanoparticles.

**Figure 7:**
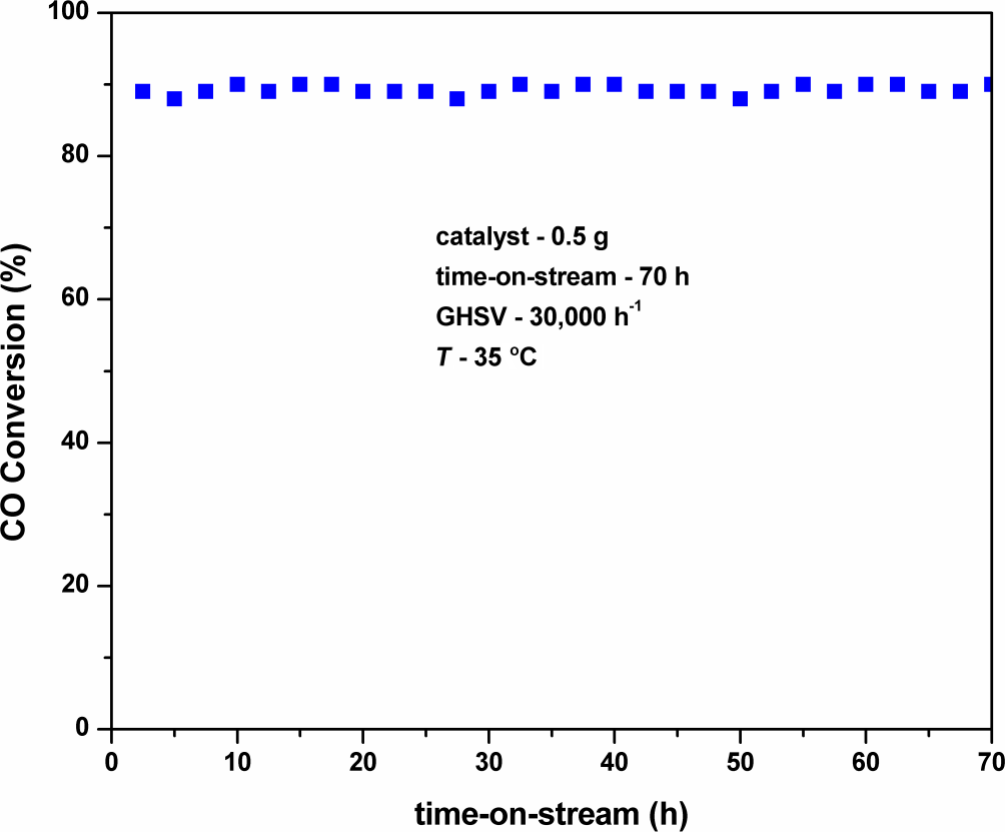
Stability test of Pt(1%)–Cu(1%)–ZrO_2_ nanoparticles during the CO oxidation reaction (catalyst: 0.5 g, CO: 500 ppm, O_2_: 20% with Ar balance, GHSV = 30,000 h^−1^, *T* = 35 °C, time-on-stream = 70 h).

The BET specific surface area of the Pt(1%)–Cu(1%)–ZrO_2_ was measured before and after the CO oxidation reaction. It was found that there was no significant change in the specific surface area. Table 3 shows the specific surface area of fresh and used Pt(1%)–Cu(1%)–ZrO_2_. This confirmed the excellent stability of the Cu/Pt co-doped sample.

**Table 3:** BET characterization of Pt(1%)–Cu(1%)–ZrO_2_.

fresh catalyst (m^2^·g^−1^)	used catalyst (m^2^·g^−1^)

65.1	64.4

### Effect of reaction conditions on CO oxidation over Pt(1%)–Cu(1%)–ZrO_2_

The effect of initial CO concentration and gas hourly space velocity (GHSV) on the CO conversion over Pt(1%)–Cu(1%)–ZrO_2_ nanoparticles is shown in Figure 8 and Figure 9. The GHSV (30,000 h^−1^) was fixed in order to observe the effect of initial concentration of CO on the conversion. With an increase in initial concentration of CO from 250 to 1000 ppm, the conversion showed a little decrease but the decrease became large as the initial concentration varied from 1000 ppm to 2000 ppm. The conversion value decreases on increasing GHSV from 15,000 h^−1^ to 60,000 h^−1^. The observed *T*_50_ value is below 40 °C at the maximum GHSV of 60,000 h^−1^.

**Figure 8:**
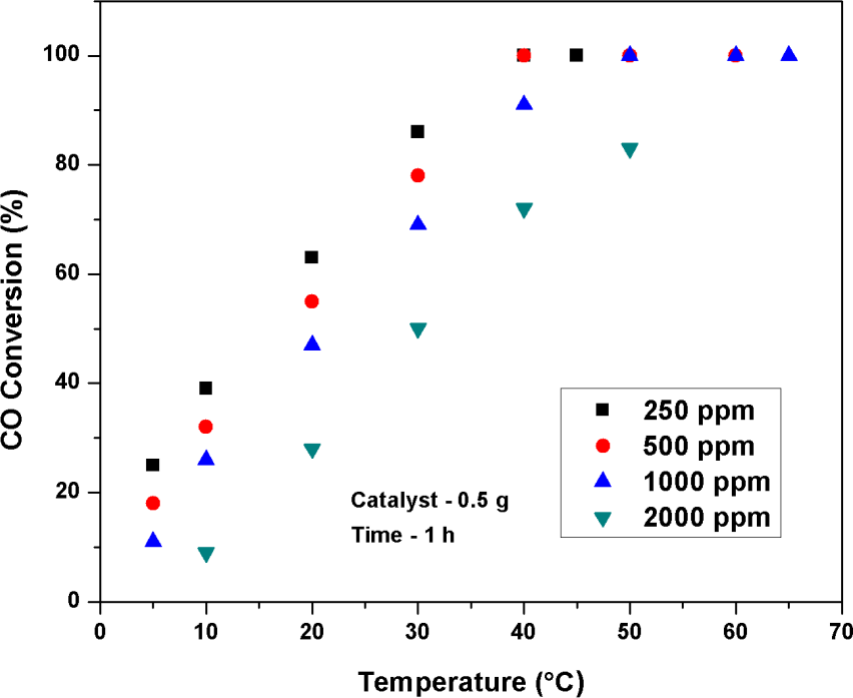
Effect of initial CO concentration on CO conversion over Pt(1%)–Cu(1%)–ZrO_2_. (Catalyst: 0.5 g, GHSV = 30,000 h^−1^, time-on-stream = 1 h).

**Figure 9:**
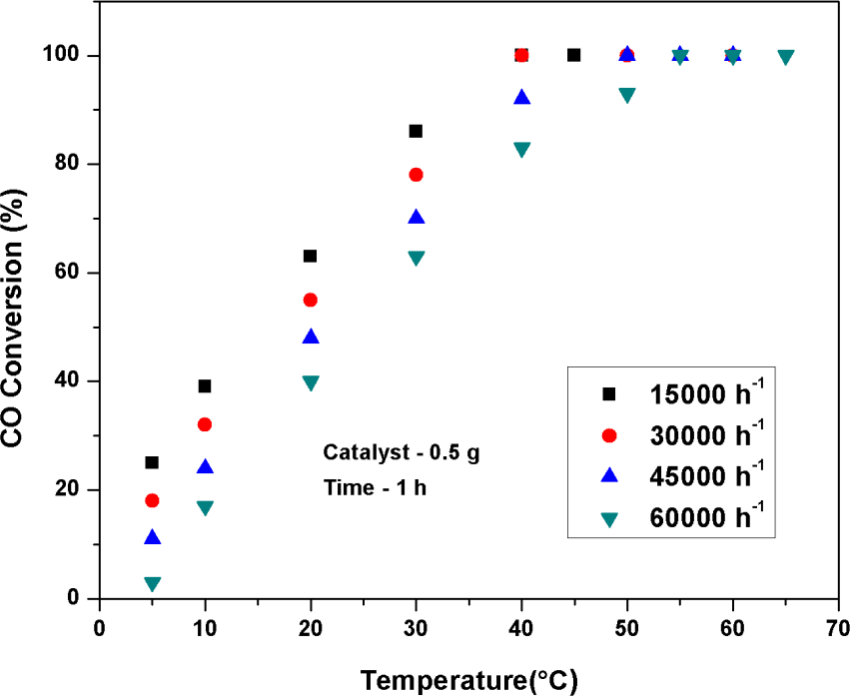
Effect of GHSV on CO conversion over Pt(1%)–Cu(1%)–ZrO_2_. (Catalyst: 0.5 g, CO: 500 ppm, O_2_: 20% with Ar balance, time-on-stream = 1 h).

## Conclusion

Pt(1%)–Cu(1%)–ZrO_2_ nanoparticles were successfully synthesized by simple solution combustion. XRD and HRTEM results revealed particles of Pt(1%)–Cu(1%)–ZrO_2_ with sizes around 10 nm. These nanoparticles were tested for CO oxidation reaction and showed a great improvement over Pt–doped ZrO_2_ and bare ZrO_2_. The *T*_50_ value was only 20 °C whereas it is 45 °C and 195 °C in Pt-doped ZrO_2_ and bare ZrO_2_, respectively. Pt(1%)–Cu(1%)–ZrO_2_ also showed excellent stability over a time-on-stream of 70 h for CO oxidation. The high catalytic activity and stability of Pt(1%)–Cu(1%)–ZrO_2_ nanoparticles is due to presence of large oxygen vacancies, high specific surface area and small particle size. This shows that the Pt/Cu co-doped ZrO_2_ is an attractive catalytic material to oxidize poisonous CO gas at very low temperatures.

## Experimental

### Material synthesis

For solution combustion [20], 0.02 mol of metal nitrates (zirconyl nitrate, hexachloroplatinic acid, copper nitrate) and 0.10 mol of urea (fuel) were taken in a beaker with 10–15 mL deionized water and mixed properly. For the synthesis of Cu/Pt co-doped ZrO_2_ nanoparticles, the fuel to metal molar ratio [urea/(Zr+Pt+Cu)] was maintained at 5 with required Zr+Pt+Cu composition. The beaker containing mixture was put into the furnace at a temperature of 400 °C for around 5 min. The solid material, Cu/Pt co-doped ZrO_2_ was produced within 5 min and was collected and calcined at 500 °C for 4 h. The sample is denoted as Pt(1%)–Cu(1%)–ZrO_2_ (the numerals indicate mol %). The amount of the Pt and Cu metals present in the catalyst sample is confirmed by ICP-AES technique (Table 1).

### Materials characterization

The powder XRD data of doped ZrO_2_ nanoparticles was performed on Rigaku X-ray diffractometer (DMAX IIIVC) instrument in a range of 2θ = 20–70°. The specific surface area of the prepared materials was measured using a BET instrument (Micrometrics, ASAP 2010). TEM analysis was done on Tecnai G^2^-20 Twin (FEI) transmission electron microscope operated at 200 kV. The synthesized nanoparticles were dispersed in 2-propanol and ultrasonicated for about 30 min and finally deposited on carbon-coated Cu grids for TEM analysis. Thermal analysis of the synthesized Cu/Pt co-doped ZrO_2_ nanoparticles was done on TGA thermal analyzer (STA-1500 Model) instrument at a heating rate of 10 °C/min in ambient atmosphere. Raman spectra (100–750 cm^−1^) were obtained on a Horiba JY LabRAM HR 800 Raman spectrometer coupled with microscope in reflectance mode with 514 nm excitation laser sources and a spectral resolution of 0.3 cm^−1^. The amount of Cu and Pt in co-doped ZrO_2_ catalysts was confirmed using a nARCOS, Simultaneous ICP-AES spectrometer.

### Catalytic activity

The CO oxidation reaction was carried out in a quartz vertical fixed-bed reactor. The prepared nanoparticles (0.5 g) were put in the quartz reactor and CO oxidation reaction was carried out at different temperatures. To simulate off-gas mixture, a mixture chamber was used in experiments. The mixture consisted of CO 500 ppm and 20% O_2_ balanced by Ar maintained at a total flow rate of 100 mL/min. Figure 10 shows the schematic of the catalyst testing activity for CO reaction. To analyze the effluent stream a gas chromatograph was used. The CO conversion was measured as follows:

[2]
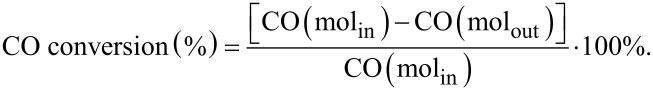


**Figure 10:**
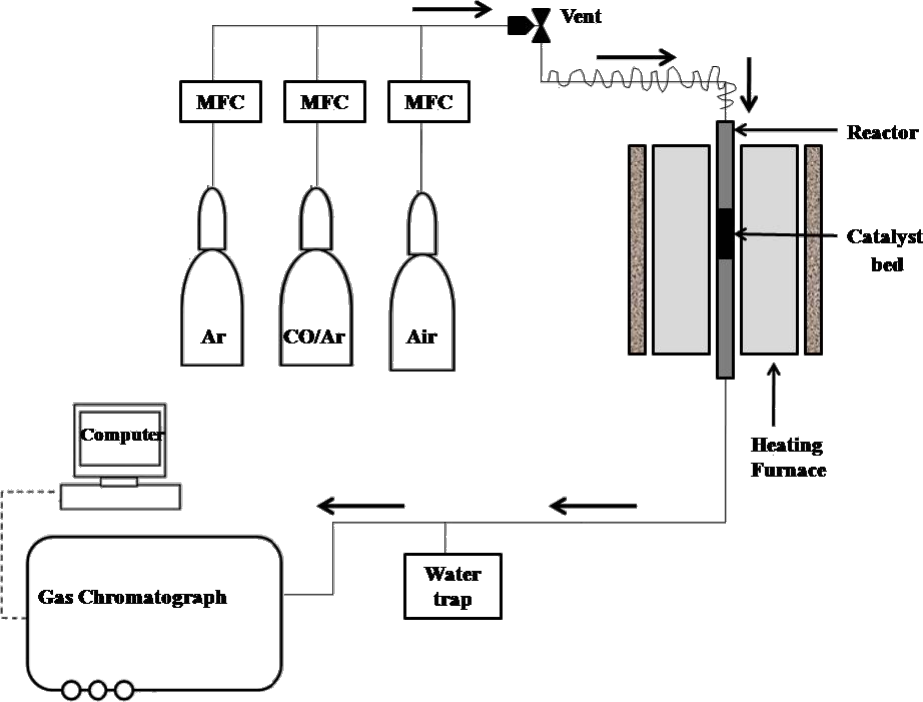
Schematic diagram of the testing of catalytic activity for CO oxidation reaction.
